# Lateral nasal osteotomy: a comparative study between the use of osteotome and a diamond surgical burr - a cadaver study

**DOI:** 10.1186/1746-160X-9-41

**Published:** 2013-12-19

**Authors:** Alireza Ghassemi, Ashraf Ayoub, Ali Modabber, Behnam Bohluli, Andreas Prescher

**Affiliations:** 1 Department of Oral, Maxillofacial and Plastic Facial Surgery, University Hospital RWTH-Aachen, Pauwelsstraße 30, 52074 Aachen, Germany; 2 MVLS College, The Dental Hospital & School, The University of Glasgow, Glasgow, United Kingdom; 3 Craniomaxillofacial Research Center, Azad University of Tehran, Tehran, Iran; 4 Institute of Anatomy, Medical Faculty of RWTH-Aachen, Aachen, Germany

**Keywords:** Lateral osteotomy, Osteotome, Diamond burr

## Abstract

**Background:**

The ultimate goal of rhinoplasty is to achieve a controllable, reliable and an aesthetically pleasing result. Various approaches and instruments have been introduced for the correction of the bony walls of the nose to improve predictability of the procedure and to minimize the associated trauma. We conducted a cadaveric study comparing the results of osteotomy of the nasal wall using a diamond surgical burr with those using a 2-mm osteotome.

**Material and methods:**

Bilateral osteotomy of the nasal wall was performed on 10 cadavers. The 20 lateral nasal osteotomies were carried out on 7 females and 3 males of an age range between 61-91 years. A 2-mm osteotome was used percutaneously to perforate the lateral nasal wall of the right side. On the left side a 2-mm diamond surgical burr was introduced via an intraoral approach to thin out the lateral nasal wall. The in-fracture of the nasal bone was accomplished by controlled finger pressure. The nasal mucosa was inspected endoscopically and also dissected to identify any perforations or lacerations. The pattern of nasal fracture and the presence of any fragmentation of the bony segments were assessed clinically.

**Results:**

The in-fracturing of the nasal bone was accomplished by gentle pressure on the left side, but required more force on the contra lateral side. On the left side the in-fractured lateral nasal wall remained as one piece and no irregularities were seen. On the right side 3-5 bony fragments of irregular sizes and shapes were detected. There were 3-4 tears of the nasal mucosa, where the osteotome was applied. However, no mucosal tears were detected at the side, where the surgical burr was used.

**Conclusion:**

Osteotomy of the lateral nasal wall with a diamond burr via intraoral approach is more precise and associated with fewer complications in comparison with the use of the osteotome.

## Introduction

Depending on the type of nasal deformities, lateral, medial, or transverse osteotomies may be indicated to improve the appearance of the nose [1,2]. The lateral nasal osteotomy is a key step in rhinoplasties and may be associated with complications, which include excessive hemorrhage, prolonged edema, ecchymosis, functional nasal obstruction from excessive narrowing, post rhinoplasty aesthetic deformity and asymmetric irregularity of the nasal wall [3]. Extensive trauma of the nasal mucosa may contribute to the prolonged postoperative ecchymosis and edema. Comminuted fracture of the nasal bones may lead to suboptimal cosmetic outcome [4].

The proper selection of surgical technique and use of appropriate instrumentation are important to achieve the desired result with low rate of complications. Various ranges of instruments have been used to improve the pattern of nasal bone fractures and minimize the associated mucosal injuries. Basically, two techniques have been used to facilitate lateral nasal osteotomy; the intranasal continuous method and the transcutaneous perforating method [5-9]. In the second method, a narrow osteotome is applied percutaneously to perforate the lateral nasal wall along the preplanned osteotomy line. This method is designed to leave the nasal mucosa and the periosteal coverage partly unaffected and to prevent the nasal bone wall from collapse [8-12]. However, it has been reported that the application of extensive force may be necessary to perforate the thick nasal bone wall [13]. Although, the method is supposed to cause less injury to the nasal mucosa, comminuted and irregular fracture lines with bony spicules have been reported [7,13,14]. In an attempt to minimize these complications a curved and guarded osteotome was suggested for performing intranasal osteotomy [15]. Nevertheless, extensive tearing of the periosteal cover and nasal mucosa has been reported [16]. To reduce these complications a narrow unguarded osteotome was used to cut through the nasal bone [7]. However, a narrow osteotome is difficult to handle in less experienced hands. For safer performance of the lateral nasal osteotomy the use of a guarded narrow osteotome was introduced to reduce the slippage. Nevertheless, damage to the surrounding soft tissue has been reported in some cases [17]. A diamond surgical burr was used to thin out the bony lateral nasal wall via an intro-oral approach along the planned osteotomy line to allow a controlled and easy in-fracturing of the bone with minimal force [18]. The results were acceptable, however, there is a lack of direct comparison between the two approaches regarding safety, reliability and associated complications.

Therefore, this cadaveric study was carried out to evaluate the shape and amount of bony fragments of the lateral nasal wall associated with each technique, and to assess the related mucosal tears.

## Material and method

Signed informed consents were obtained in lifetime for using the human bodies for scientific researches under supervision of Institute of Anatomy. The guidelines of the Helsinki Declaration have been followed for this study.

**Figure 1:**
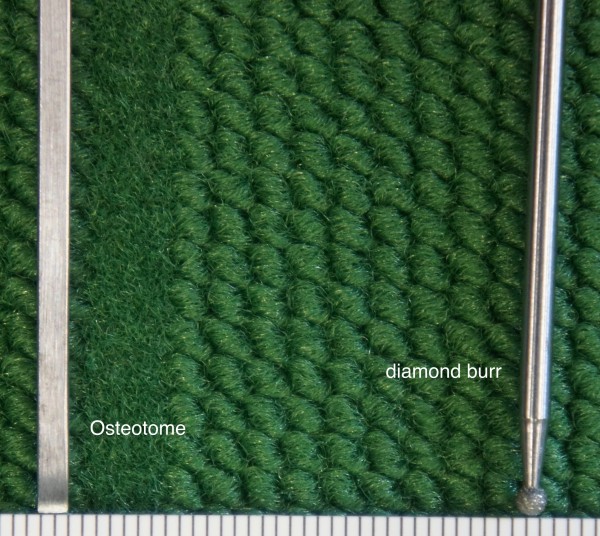
On the left side the 2-mm osteotome and on the right side the 2-mm diamond burr used for lateral nasal osteotomy.

Lateral nasal osteotomies were performed on 5 fresh and 5 formalin preserved cadavers. The age ranged from 61 to 91 years (mean age of 81), 7 males and 3 females (Table 1). Therefore, a total of 20 lateral osetomies were evaluated in this study. On the right side the lateral nasal osteotomy was performed percutaneously using a 2-mm osteotome (Aesculap AG; Tuttlingen; Germany) and on the left side a continuous cutting of the lateral wall was achieved with a 2-mm diamond surgical burr (Hager & Meisinger GmbH; Neus; Germany) (Figure 1). This was performed along a tunnel dissected by a periosteal elevator via an intra-oral approach, and guided by the osteotomy path on the skin. Finally, the nasal pyramid was in-fractured by a controlled finger pressure.

**Table 1:** The number of bone fragments and nasal mucosal tears associated with the use of an osteotome and a diamond surgical burr for the lateral nasal osteotomy

**Nr.**	**Kind of Cadaver**	**Age**	**Gender**	**Bony fragments**		**Mucosal tear**	
				**right**	**left**	**right**	**left**
1	Formalin preserved	75	f	5	1	3	0
2	Formalin preserved	77	m	4	1	4	0
3	Formalin preserved	83	f	4	1	3	0
4	Formalin preserved	87	f	6	1	2	0
5	Formalin preserved	91	f	5	1	2	0
6	Fresh cadaver	61	m	4	1	3	0
7	Fresh cadaver	76	m	3	1	3	0
8	Fresh cadaver	85	f	4	1	3	0
9	Fresh cadaver	87	f	4	1	2	0
10	Fresh cadaver	88	f	3	1	2	0
***Mean***		81		4.2	1	2.7	0
***p-value***				*p < 0,0001*		*p < 0,0001*	
±**SD**				0.92		0.67	

Blinded to the osteotomy technique, two independent examiners evaluated the result of the nasal osteotomies. The noses were inspected using endoscope (Karl Storz GmbH & CO KG, Tuttlingen; Germany) for any evidence of nasal mucosal laceration. The number of nasal lacerations associated with each method was counted. In addition, the skin and soft tissue envelope covering the nose were removed to inspect the pattern of the nasal bone fracture and evaluate any associated bony irregularities or fragmentation.

To check for statistical significance of quantitative variables Student t-test was applied (p-value of ≤ 0.05).

## Results

**Figure 2:**
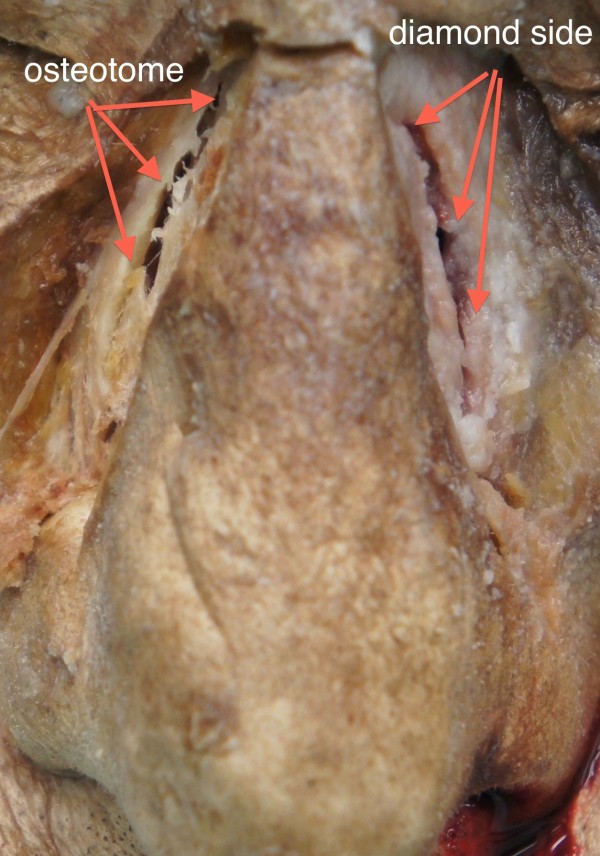
Frontal view of the performed osteotomies in fresh cadaver specimen performed on the right side with an osteotome and on the left side with diamond surgical burr.

**Figure 3:**
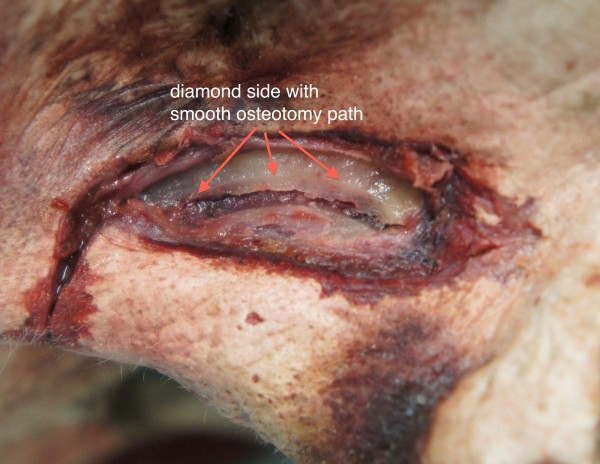
Left lateral view of the nose of a fresh cadaver specimen showing a smooth contour of its course.

**Figure 4:**
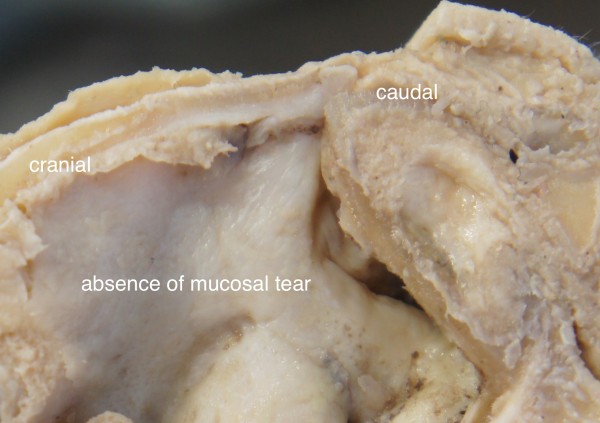
A formalin preserved cadaver nose showing absence of mucosal laceration or damage.

On the diamond side the in-fracturing of the thinned out bony wall was accomplished with a gentle finger pressure. There was a clear-cut and a smooth contour of the fracture line. The entire bony nasal wall was split in one piece with a well-defined rim (Figures 2, 3). No tearing of the nasal mucosa was detected (Figure 4).

**Figure 5:**
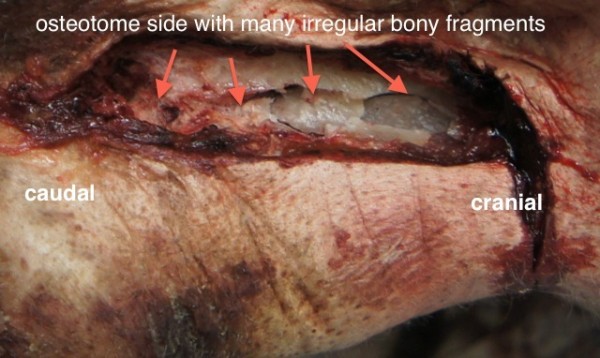
Right lateral view of the nose of a fresh cadaver specimen showing many irregular fragments along the course of the bone cut.

**Figure 6:**
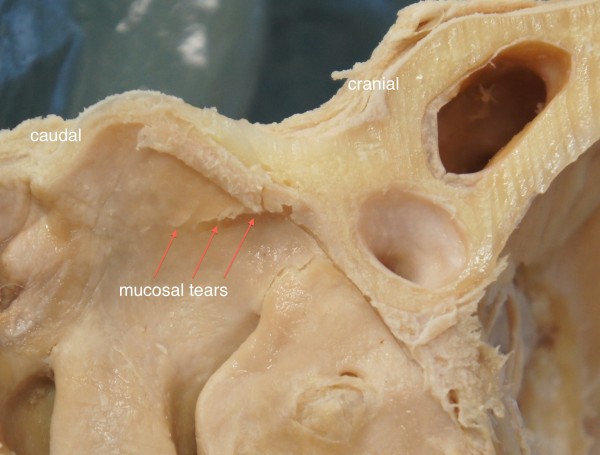
Perforation of the nasal mucosa along the osteotomy path as a result of use of osteotome for bone cut.

On the contrary, more force was needed to in-fracture the lateral nasal bony wall on the osteotome side. In 8 out of 10 lateral nasal walls more than one attempt was required to weaken the nasal bone for in-fracturing. We observed 3-5 comminuted fracture lines with small bony fragments of irregular shapes and sizes (Table 1, Figures 2, 5). Additionally, 2-4 tears of the nasal mucosa were found (Figure 6). There were statistical significant differences regarding the number of bone fragmentations (p < 0.0001; SD 0.92) and mucosal tears (p < 0.0001; SD 0.67) between the two sides of the nose. These were significantly higher on the osteotome side.

## Discussion

The ideal technique for lateral nasal osteotomy should maintain the integrity of the nasal mucosa and the periosteal attachment of the bony lateral wall of the nose. The method should be safe, precise, reproducible and do not cause irregularities of the lateral nasal wall [1,2].

The purpose of this study was to assess the incidence and degree of the nasal mucosal tearing produced by an osteotome compared to that caused by a diamond surgical burr for cutting the lateral nasal wall. It was also our objective to analyze the shape, size and amount of associated fragmentation of the bony nasal wall, which may influence the long-term esthetic outcome of the procedure.

Two approaches are used to facilitate the lateral nasal osteotomy; the transcutanous and the endonasal approaches [9-13]. The transcutanous approach is claimed to cause minimal mucosal tear and allow a good control of osteotomy procedure [12]. However, the force required to perforate the lateral bony wall of the nose could cause irregular fracture patterns, and fragmentation bony wall, which would lead to dorsum irregularities [13,19]. The other option is the continuous method, which is generally performed via an intranasal approach using an osteotome, which eliminates the need for skin incision. This method promises fewer episodes of comminuted fracture patterns of the nasal bone and consequently reduces the chance for postoperative nasal irregularities. However, this method may cause extensive mucosal tear, which could be reduced if the bone cut is achieved via the same approach using a smaller osteotome of 3 mm width [7]. On the other hand, it is difficult to handle this small osteotome, especially in less experienced hands and therefore using a guarded osteotome may reduce the tearing of the nasal mucosa [9,17]. Nevertheless, the method is complex and requires extensive manipulation to perforate or cut through the lateral nasal wall along the planned osteotomy line.

To reduce the risk for comminuted fractures of the nasal bone or lacerations of the nasal mucosa, we introduced the concept of lateral nasal wall osteotomy using a diamond surgical burr via an intraoral approach. We observed a clean osteotomy cut using this method and the in-fracture of the nasal bone was readily achieved with gentle finger pressure. This anatomical study confirmed the finding observed clinically before [18]. The use of a diamond surgical burr was a reliable and safe method to perform the ostetotomy of bony nasal wall. With the use of diamond surgical burr there was no need to cut the lateral nasal wall completely and a thinning of the nasal bone was sufficient to in-fracture the nasal walls in a reliable and predictable fashion. The nasal mucosa remained intact, the lateral nasal wall split in one piece without comminuted fracture. The disadvantage of this method is the need for an additional intraoral incision to approach the pyriform aperture; however, this allows the cut of a smooth osteotomy line with less fragmentation and bony irregularities. This is essential clinically since unfavorable fracture line of the nasal bones would lead to unsightly contour irregularity and palpable bone spur. The presented intra oral approach offers a high degree of controllability, reproducibility and minimal complications. It is associated with a reduced risk of soft tissue injury and facilitates the clean split of the nasal bone. We acknowledge the limitation of this study, which was carried out on cadavers. The clinical impact of the lacerations of the nasal mucosa that were associated with the osteotome cut cannot be investigated exactly. This research prepares the way for a clinical prospective randomized study to investigate the clinical advantages of the newly introduced technique. We also appreciate that other types and sizes of surgical osteotomes may require further investigation. Nevertheless, this study tested the new approach, which compared favourably with the gold standard method of using osteotome for lateral nasal osteotomy.

## Conclusion

The use of diamond surgical burr for guided lateral nasal wall osteotomy is a reliable technique; it facilitates the uncomplicated in-fracture of the bony segments with minimal fragmentation and a limited damage to the surrounding soft tissues.

## Competing interests

The authors declare that they have no competing interests.

## Authors’ contributions

AG, AA, AM, BB and AP conceived of the study and participated in its design and coordination. AG, AM, AP made substantial contributions to literature review, data acquisition and conception of manuscript. AG and AM conducted statistical analysis and drafted the manuscript. AA, BB and AP were involved in revising the manuscript. All authors read and approved the final manuscript.
